# Chlorido(dimethyl sulfoxide-κ*O*)triphenyl­tin(IV)

**DOI:** 10.1107/S1600536809048090

**Published:** 2009-11-18

**Authors:** Sarvendra Kumar, Shah Mohammad Shadab, Mohammad Idrees

**Affiliations:** a Indian Institute of Technology, Kanpur, India; b Department of Chemical Engineering, Aligarh Muslim University, Aligarh, India

## Abstract

In the title compound, [Sn(C_6_H_5_)_3_Cl(C_2_H_6_OS)], the Sn^IV^ atom is coordinated by three phenyl groups, a chloride ion and a dimethyl sulfoxide mol­ecule in a distorted trigonal-bipyramidal geometry. In the crystal, adjacent mol­ecules are linked through inter­molecular C—H⋯Cl hydrogen bonds, weak C—H⋯π inter­actions and π–π inter­actions [centroid–centroid distance = 3.934 (3) Å. An intra­molecular C—H⋯π inter­action is also observed.

## Related literature

For general background to the biological activity and industrial applications of triorganotin(IV) complexes, see: Willem *et al.* (1997 ▶); Gielen *et al.* (2000 ▶); Tian *et al.* (2005 ▶). For bond-length data, see: Allen *et al.* (1987 ▶). For some unusual examples of [Sn(C_6_H_5_)_3_(C_16_H_10_NO_3_)(C_2_H_6_O)] adducts with oxygen-donor ligands, see: Lo & Ng (2009 ▶); Ng & Kumar Das (1997 ▶).
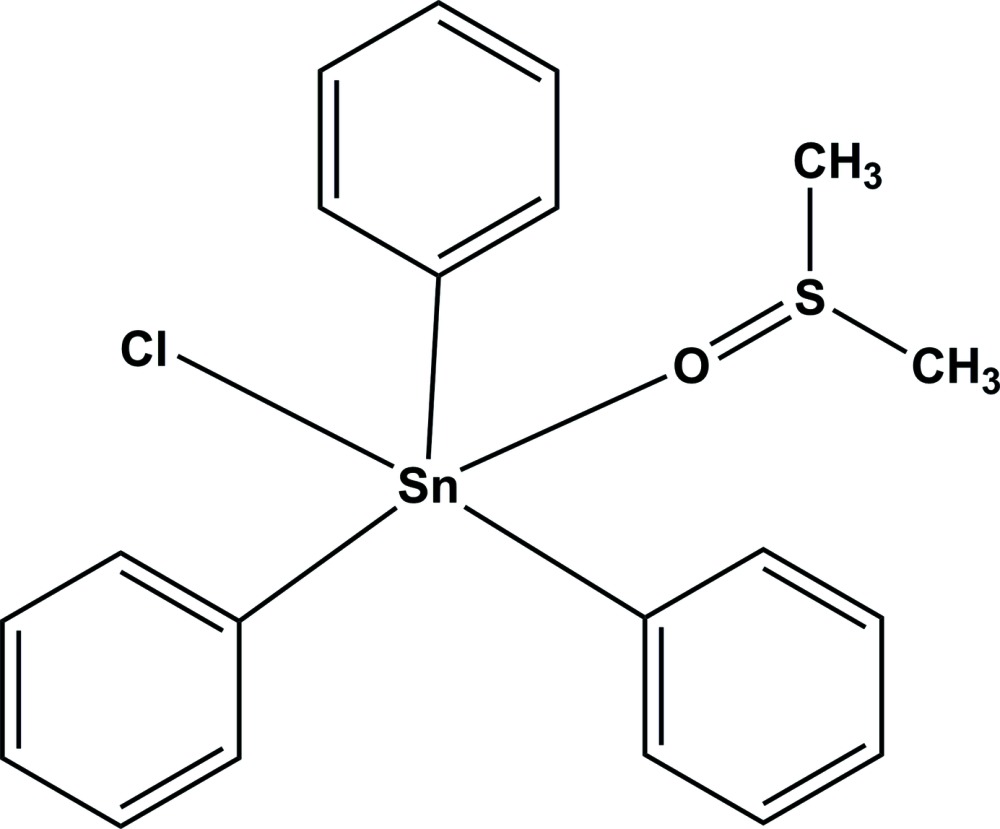


## Experimental

### 

#### Crystal data

[Sn(C_6_H_5_)_3_Cl(C_2_H_6_OS)]
*M* *_r_* = 463.57
Orthorhombic, 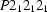

*a* = 10.417 (5) Å
*b* = 13.235 (5) Å
*c* = 14.302 (5) Å
*V* = 1971.8 (14) Å^3^
*Z* = 4
Mo *K*α radiation
μ = 1.54 mm^−1^
*T* = 293 K
0.26 × 0.24 × 0.22 mm

#### Data collection

Bruker SMART CCD diffractometer
Absorption correction: multi-scan (**SADABS**; Sheldrick, 2004 ▶) *T*_min_ = 0.677, *T*_max_ = 0.712
11203 measured reflections
4052 independent reflections
3877 reflections with *I* > 2σ(*I*)
*R*_int_ = 0.048

#### Refinement

*R*[*F* ^2^ > 2σ(*F* ^2^)] = 0.031
*wR*(*F* ^2^) = 0.085
*S* = 1.15
4052 reflections
217 parameters
H-atom parameters constrained
Δρ_max_ = 1.18 e Å^−3^
Δρ_min_ = −0.86 e Å^−3^
Absolute structure: Flack (1983 ▶), 1732 Friedel pairs
Flack parameter: −0.07 (3)

### 

Data collection: *SMART* (Bruker, 2001 ▶); cell refinement: *SAINT* (Bruker, 2001 ▶); data reduction: *SAINT*; program(s) used to solve structure: *SHELXS97* (Sheldrick, 2008 ▶); program(s) used to refine structure: *SHELXL97* (Sheldrick, 2008 ▶); molecular graphics: *SHELXTL* (Sheldrick, 2008 ▶); software used to prepare material for publication: *SHELXTL*.

## Supplementary Material

Crystal structure: contains datablocks I, global. DOI: 10.1107/S1600536809048090/is2466sup1.cif

Structure factors: contains datablocks I. DOI: 10.1107/S1600536809048090/is2466Isup2.hkl

Additional supplementary materials:  crystallographic information; 3D view; checkCIF report

## Figures and Tables

**Table 1:** Selected bond lengths (Å)

Sn1—Cl1	2.4999 (14)
Sn1—O1	2.311 (3)
Sn1—C1	2.134 (5)
Sn1—C7	2.132 (5)
Sn1—C13	2.131 (5)

**Table 2:** Hydrogen-bond geometry (Å, °)

*D*—H⋯*A*	*D*—H	H⋯*A*	*D*⋯*A*	*D*—H⋯*A*
C20—H20*C*⋯Cl1^i^	0.96	2.69	3.610 (6)	161
C20—H20*A*⋯*Cg*3	0.96	2.94	3.813 (6)	151
C20—H20*B*⋯*Cg*1^ii^	0.96	2.61	3.490 (6)	153

Symmetry codes: (i) 
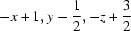
; (ii) 
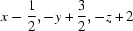
. *Cg*1 and *Cg*3 are the centroids of the C1–C6 and C13–C18 rings, respectively.

**Fig. 1.**
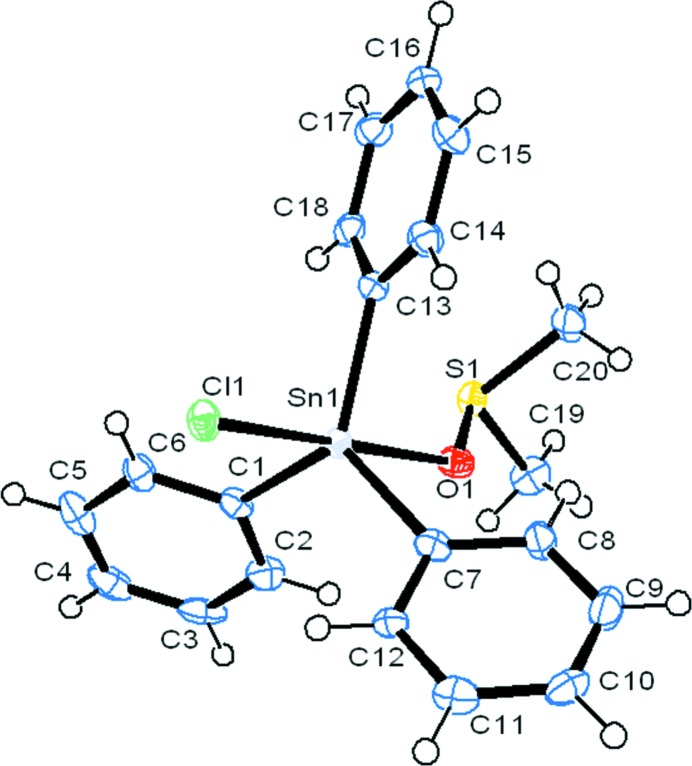
An ORTEP diagram of the title compound, with displacement ellipsoids drawn at the 50% probability level for non-hydrogen atoms.

**Fig. 2.**
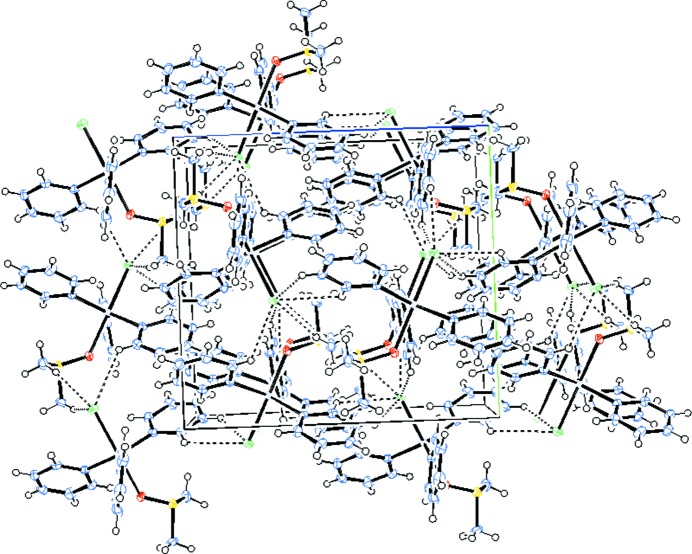
In the packing diagram, molecules are bounded by weak intermolecular weak hydrogen bonds.

## supplementary crystallographic information

### Comment

Triorganotin(IV) complexes are well known for their biological activities as well as industrial applications (Willem *et al.*, 1997; Gielen *et al.*, 2000; Tian *et al.*, 2005). Owing to wide spread applications of organotin compounds, their synthesis and characterization with O–containing ligands have been a continuing subject in recent years (Lo & Ng, 2009, Ng & Kumar Das, 1997). There are very rare examples of the triorganotin(IV) complexes with solvent molecules (Lo & Ng, 2009), in which solvent molecule acts as a chelator ligand.

The bond lengths and bond angles in the molecules are within normal ranges (Allen *et al.* 1987). The Sn^IV^ atom is coordinated by three phenyl groups, one chloride ion and one solvent molecule (DMSO) in a distorted trigonal biyramidal geometry (Fig. 1). The three phenyl groups are attached in a plane to the Sn atom and seemed like as three pedal of ceiling fan. The one chloride and one solvent molecule are located at axial positions and three phenyl groups are located at equatorial positions. Each phenyl group of the one compound is interacted with another phenyl group of another molecule by a π–π interaction and further interacted by a C—H···π interaction. The neighboring molecules are bound by C—H···Cl, C—H···π (Table 2) and a π–π interaction with a centroid-centroid distance of 3.934 (3) Å (Fig. 2).

### Experimental

Triphenyltinchloride (0.385 g, 1 mmol) was dissolved in DMSO (4 ml) and heated until the reactant dissolved completely. The solution was filtered and solvent allowed evaporating slowly. Fine colorless crystal produced after 3 days. Crystals are stable at room temperature. Analysis calc. for C_20_H_21_SOClSn: C 51.81, H 4.57, S 6.92. Found: C 51.68, H 4.56, S 6.95.

### Refinement

All of the hydrogen atoms were placed in calculated positions (C–H = 0.93 or 0.96 Å) and were included in the refinement in the riding model approximation, with *U*_iso_(H) set to 1.2*U*_eq_(C). The highest residual electron density peak is located 0.87 Å from atom Sn1. The Hooft parameter value is -0.045 (17).

### Crystal data

**Table:**

[Sn(C_6_H_5_)_3_Cl(C_2_H_6_OS)]	*F*(000) = 928
*M**_r_* = 463.57	*D*_x_ = 1.562 Mg m^−^^3^
Orthorhombic, *P*2_1_2_1_2_1_	Mo *K*α radiation, λ = 0.71069 Å
Hall symbol: P 2ac 2ab	Cell parameters from 6343 reflections
*a* = 10.417 (5) Å	θ = 2.4–28.3°
*b* = 13.235 (5) Å	µ = 1.54 mm^−^^1^
*c* = 14.302 (5) Å	*T* = 293 K
*V* = 1971.8 (14) Å^3^	Prism, colorless
*Z* = 4	0.26 × 0.24 × 0.22 mm

### Data collection

**Table:**

Bruker SMART CCD diffractometer	4052 independent reflections
Radiation source: fine-focus sealed tube	3877 reflections with *I* > 2σ(*I*)
graphite	*R*_int_ = 0.048
φ and ω scans	θ_max_ = 26.5°, θ_min_ = 2.1°
Absorption correction: multi-scan (*SADABS*; Sheldrick, 2004)	*h* = −13→12
*T*_min_ = 0.677, *T*_max_ = 0.712	*k* = −16→15
11203 measured reflections	*l* = −15→17

### Refinement

**Table:**

Refinement on *F*^2^	Secondary atom site location: difference Fourier map
Least-squares matrix: full	Hydrogen site location: inferred from neighbouring sites
*R*[*F*^2^ > 2σ(*F*^2^)] = 0.031	H-atom parameters constrained
*wR*(*F*^2^) = 0.085	*w* = 1/[σ^2^(*F*_o_^2^) + (0.0344*P*)^2^ + 2.3774*P*] where *P* = (*F*_o_^2^ + 2*F*_c_^2^)/3
*S* = 1.15	(Δ/σ)_max_ < 0.001
4052 reflections	Δρ_max_ = 1.18 e Å^−^^3^
217 parameters	Δρ_min_ = −0.86 e Å^−^^3^
0 restraints	Absolute structure: Flack (1983), 1732 Friedel pairs
Primary atom site location: structure-invariant direct methods	Flack parameter: −0.07 (3)

### Special details

**Table:**

Geometry. All e.s.d.'s (except the e.s.d. in the dihedral angle between two l.s. planes) are estimated using the full covariance matrix. The cell e.s.d.'s are taken into account individually in the estimation of e.s.d.'s in distances, angles and torsion angles; correlations between e.s.d.'s in cell parameters are only used when they are defined by crystal symmetry. An approximate (isotropic) treatment of cell e.s.d.'s is used for estimating e.s.d.'s involving l.s. planes.
Refinement. Refinement of *F*^2^ against ALL reflections. The weighted *R*-factor *wR* and goodness of fit *S* are based on *F*^2^, conventional *R*-factors *R* are based on *F*, with *F* set to zero for negative *F*^2^. The threshold expression of *F*^2^ > σ(*F*^2^) is used only for calculating *R*-factors(gt) *etc*. and is not relevant to the choice of reflections for refinement. *R*-factors based on *F*^2^ are statistically about twice as large as those based on *F*, and *R*- factors based on ALL data will be even larger.

### Fractional atomic coordinates and isotropic or equivalent isotropic displacement parameters (Å^2^)

**Table:**

	*x*	*y*	*z*	*U*_iso_*/*U*_eq_	
Sn1	0.65650 (3)	0.90124 (2)	0.76810 (2)	0.01581 (9)	
Cl1	0.70946 (11)	1.06792 (8)	0.69548 (9)	0.0215 (2)	
S1	0.60492 (11)	0.71709 (9)	0.93706 (8)	0.0203 (2)	
O1	0.6043 (3)	0.7472 (2)	0.8339 (2)	0.0212 (7)	
C17	0.5280 (5)	1.0132 (4)	1.0442 (4)	0.0253 (11)	
H17	0.5586	1.0112	1.1053	0.030*	
C14	0.4365 (4)	1.0183 (4)	0.8606 (4)	0.0227 (10)	
H14	0.4060	1.0203	0.7995	0.027*	
C3	1.0216 (5)	0.7334 (4)	0.7804 (3)	0.0295 (12)	
H3	1.0461	0.6662	0.7735	0.035*	
C2	0.8928 (5)	0.7602 (4)	0.7732 (4)	0.0246 (10)	
H2	0.8319	0.7104	0.7614	0.030*	
C9	0.3759 (5)	0.7531 (4)	0.5849 (4)	0.0287 (12)	
H9	0.3023	0.7144	0.5939	0.034*	
C18	0.5971 (5)	0.9694 (3)	0.9717 (3)	0.0211 (10)	
H18	0.6751	0.9384	0.9851	0.025*	
C13	0.5538 (4)	0.9702 (3)	0.8800 (3)	0.0179 (9)	
C12	0.5932 (5)	0.8685 (4)	0.5590 (3)	0.0198 (10)	
H12	0.6661	0.9078	0.5497	0.024*	
C8	0.4438 (4)	0.7872 (4)	0.6612 (4)	0.0227 (10)	
H8	0.4163	0.7705	0.7211	0.027*	
C7	0.5534 (4)	0.8467 (3)	0.6496 (3)	0.0185 (10)	
C19	0.6133 (5)	0.5836 (4)	0.9315 (4)	0.0279 (11)	
H19A	0.6975	0.5636	0.9114	0.042*	
H19B	0.5506	0.5590	0.8878	0.042*	
H19C	0.5963	0.5557	0.9922	0.042*	
C10	0.4154 (5)	0.7756 (4)	0.4954 (4)	0.0256 (11)	
H10	0.3687	0.7527	0.4443	0.031*	
C20	0.4428 (5)	0.7303 (4)	0.9743 (4)	0.0265 (11)	
H20A	0.4226	0.8007	0.9809	0.040*	
H20B	0.4316	0.6969	1.0333	0.040*	
H20C	0.3868	0.7004	0.9287	0.040*	
C11	0.5260 (5)	0.8328 (4)	0.4823 (4)	0.0249 (11)	
H11	0.5547	0.8470	0.4221	0.030*	
C4	1.1127 (5)	0.8061 (4)	0.7976 (4)	0.0310 (12)	
H4	1.1988	0.7883	0.8023	0.037*	
C6	0.9465 (4)	0.9331 (4)	0.8018 (4)	0.0233 (10)	
H6	0.9223	1.0002	0.8101	0.028*	
C1	0.8536 (4)	0.8603 (3)	0.7833 (3)	0.0187 (9)	
C16	0.4115 (5)	1.0605 (4)	1.0232 (4)	0.0262 (11)	
H16	0.3640	1.0906	1.0706	0.031*	
C5	1.0761 (5)	0.9062 (5)	0.8081 (4)	0.0313 (12)	
H5	1.1378	0.9556	0.8192	0.038*	
C15	0.3661 (5)	1.0628 (4)	0.9314 (4)	0.0293 (12)	
H15	0.2884	1.0942	0.9179	0.035*	

### Atomic displacement parameters (Å^2^)

**Table:**

	*U*^11^	*U*^22^	*U*^33^	*U*^12^	*U*^13^	*U*^23^
Sn1	0.01343 (14)	0.01702 (14)	0.01697 (15)	0.00044 (12)	0.00008 (12)	0.00061 (12)
Cl1	0.0183 (5)	0.0193 (5)	0.0268 (6)	−0.0005 (4)	−0.0006 (5)	0.0046 (4)
S1	0.0192 (5)	0.0212 (6)	0.0205 (6)	−0.0035 (5)	−0.0025 (5)	0.0022 (5)
O1	0.0262 (16)	0.0221 (17)	0.0152 (16)	0.0020 (14)	0.0026 (14)	0.0018 (13)
C17	0.033 (3)	0.023 (2)	0.020 (3)	−0.004 (2)	0.003 (2)	−0.001 (2)
C14	0.018 (2)	0.025 (2)	0.025 (3)	0.002 (2)	0.000 (2)	−0.001 (2)
C3	0.030 (3)	0.039 (3)	0.020 (3)	0.020 (2)	0.000 (2)	−0.007 (2)
C2	0.022 (2)	0.028 (2)	0.025 (2)	0.0069 (19)	−0.004 (2)	−0.002 (2)
C9	0.020 (3)	0.026 (3)	0.040 (3)	−0.002 (2)	−0.006 (2)	−0.006 (2)
C18	0.021 (2)	0.014 (2)	0.029 (3)	−0.0012 (19)	0.000 (2)	−0.001 (2)
C13	0.018 (2)	0.019 (2)	0.017 (2)	−0.0021 (19)	0.0027 (19)	−0.0019 (18)
C12	0.021 (2)	0.023 (2)	0.015 (2)	−0.0004 (19)	0.0012 (19)	−0.0036 (18)
C8	0.017 (2)	0.025 (2)	0.026 (3)	−0.002 (2)	0.001 (2)	0.000 (2)
C7	0.016 (2)	0.018 (2)	0.022 (2)	0.0074 (18)	−0.0002 (19)	0.0010 (19)
C19	0.035 (3)	0.020 (3)	0.028 (3)	0.004 (2)	−0.004 (2)	0.006 (2)
C10	0.029 (3)	0.025 (3)	0.024 (3)	0.005 (2)	−0.009 (2)	−0.008 (2)
C20	0.027 (3)	0.036 (3)	0.017 (2)	−0.007 (2)	0.002 (2)	0.002 (2)
C11	0.033 (3)	0.022 (2)	0.020 (2)	0.006 (2)	0.003 (2)	0.0051 (19)
C4	0.021 (2)	0.052 (3)	0.020 (2)	0.012 (2)	0.001 (2)	0.004 (2)
C6	0.017 (2)	0.029 (3)	0.024 (2)	0.0002 (19)	−0.001 (2)	0.003 (2)
C1	0.015 (2)	0.025 (2)	0.016 (2)	0.0042 (18)	−0.001 (2)	0.0016 (17)
C16	0.024 (3)	0.029 (3)	0.025 (3)	−0.005 (2)	0.005 (2)	−0.011 (2)
C5	0.018 (2)	0.046 (3)	0.029 (3)	−0.001 (2)	0.003 (2)	0.010 (3)
C15	0.017 (3)	0.033 (3)	0.038 (3)	0.002 (2)	0.003 (2)	−0.005 (2)

### Geometric parameters (Å, °)

**Table:**

Sn1—Cl1	2.4999 (14)	C12—C11	1.384 (7)
Sn1—O1	2.311 (3)	C12—C7	1.391 (7)
Sn1—C1	2.134 (5)	C12—H12	0.9300
Sn1—C7	2.132 (5)	C8—C7	1.397 (7)
Sn1—C13	2.131 (5)	C8—H8	0.9300
S1—O1	1.529 (3)	C19—H19A	0.9600
S1—C19	1.771 (5)	C19—H19B	0.9600
S1—C20	1.779 (5)	C19—H19C	0.9600
C17—C18	1.389 (7)	C10—C11	1.391 (7)
C17—C16	1.399 (8)	C10—H10	0.9300
C17—H17	0.9300	C20—H20A	0.9600
C14—C15	1.382 (7)	C20—H20B	0.9600
C14—C13	1.405 (7)	C20—H20C	0.9600
C14—H14	0.9300	C11—H11	0.9300
C3—C4	1.374 (8)	C4—C5	1.387 (8)
C3—C2	1.391 (7)	C4—H4	0.9300
C3—H3	0.9300	C6—C1	1.392 (7)
C2—C1	1.394 (6)	C6—C5	1.398 (7)
C2—H2	0.9300	C6—H6	0.9300
C9—C10	1.377 (8)	C16—C15	1.395 (8)
C9—C8	1.376 (7)	C16—H16	0.9300
C9—H9	0.9300	C5—H5	0.9300
C18—C13	1.387 (7)	C15—H15	0.9300
C18—H18	0.9300		
			
C13—Sn1—C7	119.29 (18)	C7—C8—H8	119.6
C13—Sn1—C1	121.03 (17)	C12—C7—C8	118.1 (4)
C7—Sn1—C1	118.63 (17)	C12—C7—Sn1	121.4 (3)
C13—Sn1—O1	87.39 (15)	C8—C7—Sn1	120.6 (4)
C7—Sn1—O1	84.62 (15)	S1—C19—H19A	109.5
C1—Sn1—O1	87.74 (15)	S1—C19—H19B	109.5
C13—Sn1—Cl1	92.56 (13)	H19A—C19—H19B	109.5
C7—Sn1—Cl1	94.58 (13)	S1—C19—H19C	109.5
C1—Sn1—Cl1	93.10 (12)	H19A—C19—H19C	109.5
O1—Sn1—Cl1	179.04 (9)	H19B—C19—H19C	109.5
O1—S1—C19	102.5 (2)	C9—C10—C11	119.4 (5)
O1—S1—C20	105.1 (2)	C9—C10—H10	120.3
C19—S1—C20	99.1 (3)	C11—C10—H10	120.3
S1—O1—Sn1	128.46 (19)	S1—C20—H20A	109.5
C18—C17—C16	118.4 (5)	S1—C20—H20B	109.5
C18—C17—H17	120.8	H20A—C20—H20B	109.5
C16—C17—H17	120.8	S1—C20—H20C	109.5
C15—C14—C13	120.7 (5)	H20A—C20—H20C	109.5
C15—C14—H14	119.7	H20B—C20—H20C	109.5
C13—C14—H14	119.7	C12—C11—C10	119.8 (5)
C4—C3—C2	120.1 (5)	C12—C11—H11	120.1
C4—C3—H3	120.0	C10—C11—H11	120.1
C2—C3—H3	120.0	C3—C4—C5	119.9 (5)
C3—C2—C1	121.2 (5)	C3—C4—H4	120.1
C3—C2—H2	119.4	C5—C4—H4	120.1
C1—C2—H2	119.4	C1—C6—C5	120.5 (5)
C10—C9—C8	120.8 (5)	C1—C6—H6	119.8
C10—C9—H9	119.6	C5—C6—H6	119.8
C8—C9—H9	119.6	C6—C1—C2	118.3 (4)
C13—C18—C17	122.3 (5)	C6—C1—Sn1	120.8 (3)
C13—C18—H18	118.8	C2—C1—Sn1	120.9 (3)
C17—C18—H18	118.8	C15—C16—C17	120.4 (5)
C18—C13—C14	118.2 (4)	C15—C16—H16	119.8
C18—C13—Sn1	122.9 (3)	C17—C16—H16	119.8
C14—C13—Sn1	118.9 (3)	C4—C5—C6	120.1 (5)
C11—C12—C7	121.1 (5)	C4—C5—H5	119.9
C11—C12—H12	119.4	C6—C5—H5	119.9
C7—C12—H12	119.4	C14—C15—C16	120.0 (5)
C9—C8—C7	120.7 (5)	C14—C15—H15	120.0
C9—C8—H8	119.6	C16—C15—H15	120.0
			
C19—S1—O1—Sn1	−159.5 (3)	Cl1—Sn1—C7—C12	37.2 (4)
C20—S1—O1—Sn1	97.4 (3)	C13—Sn1—C7—C8	−47.5 (4)
C13—Sn1—O1—S1	−41.8 (3)	C1—Sn1—C7—C8	120.9 (4)
C7—Sn1—O1—S1	−161.5 (3)	O1—Sn1—C7—C8	36.4 (4)
C1—Sn1—O1—S1	79.4 (3)	Cl1—Sn1—C7—C8	−143.1 (4)
C4—C3—C2—C1	−0.1 (8)	C8—C9—C10—C11	0.5 (8)
C16—C17—C18—C13	−0.6 (7)	C7—C12—C11—C10	1.2 (7)
C17—C18—C13—C14	0.7 (7)	C9—C10—C11—C12	−1.5 (7)
C17—C18—C13—Sn1	−178.9 (4)	C2—C3—C4—C5	0.2 (8)
C15—C14—C13—C18	−0.5 (7)	C5—C6—C1—C2	1.4 (7)
C15—C14—C13—Sn1	179.1 (4)	C5—C6—C1—Sn1	−177.5 (4)
C7—Sn1—C13—C18	152.0 (4)	C3—C2—C1—C6	−0.7 (8)
C1—Sn1—C13—C18	−16.1 (5)	C3—C2—C1—Sn1	178.2 (4)
O1—Sn1—C13—C18	69.7 (4)	C13—Sn1—C1—C6	−61.1 (4)
Cl1—Sn1—C13—C18	−111.3 (4)	C7—Sn1—C1—C6	130.6 (4)
C7—Sn1—C13—C14	−27.5 (4)	O1—Sn1—C1—C6	−146.7 (4)
C1—Sn1—C13—C14	164.3 (3)	Cl1—Sn1—C1—C6	33.7 (4)
O1—Sn1—C13—C14	−109.9 (4)	C13—Sn1—C1—C2	120.0 (4)
Cl1—Sn1—C13—C14	69.2 (4)	C7—Sn1—C1—C2	−48.2 (4)
C10—C9—C8—C7	0.9 (8)	O1—Sn1—C1—C2	34.4 (4)
C11—C12—C7—C8	0.2 (7)	Cl1—Sn1—C1—C2	−145.1 (4)
C11—C12—C7—Sn1	179.9 (4)	C18—C17—C16—C15	0.3 (8)
C9—C8—C7—C12	−1.3 (7)	C3—C4—C5—C6	0.5 (8)
C9—C8—C7—Sn1	179.0 (4)	C1—C6—C5—C4	−1.3 (8)
C13—Sn1—C7—C12	132.8 (4)	C13—C14—C15—C16	0.2 (8)
C1—Sn1—C7—C12	−58.8 (4)	C17—C16—C15—C14	−0.2 (8)
O1—Sn1—C7—C12	−143.3 (4)		

### Hydrogen-bond geometry (Å, °)

**Table:**

*D*—H···*A*	*D*—H	H···*A*	*D*···*A*	*D*—H···*A*
C20—H20C···Cl1^i^	0.96	2.69	3.610 (6)	161
C20—H20A···Cg3	0.96	2.94	3.813 (6)	151
C20—H20B···Cg1^ii^	0.96	2.61	3.490 (6)	153

Symmetry codes: (i) −*x*+1, *y*−1/2, −*z*+3/2; (ii) *x*−1/2, −*y*+3/2, −*z*+2.
